# Can Acupuncture Treatment of Hypertension Improve Brain Health? A Mini Review

**DOI:** 10.3389/fnagi.2019.00240

**Published:** 2019-09-13

**Authors:** Jongjoo Sun, John Ashley, J. Mikhail Kellawan

**Affiliations:** Human Circulation Research Laboratory, Department of Health and Exercise Science, University of Oklahoma, Norman, OK, United States

**Keywords:** acupuncture, hypertension, cerebral blood flow, cerebrovascular disease, neurodegenerative disease, blood pressure

## Abstract

With age, cerebrovascular and neurodegenerative diseases (e.g., dementia and Alzheimer’s) are some of the leading causes of death in the United States. Related to these outcomes is the increased prevalence of hypertension, which independently increases the development of cerebrovascular and neurodegenerative diseases. While a direct mechanistic link between hypertension and poor brain health is unknown, many hypothesize that the etiology stems from poor blood pressure (BP) and cerebrovascular regulation. This dysfunction fosters hypoperfusion of the brain, causing stress to the tissue through a nutrient mismatch, subtly damaging the brain over many years. Current Western medical treatment relies on pharmacological treatment (mainly beta-blockers, angiotensin-converting enzyme inhibitors, or a combination of the two). However, Western treatments have not been successful in mitigating brain health outcomes and are burdened with unwanted side effects and non-adherence issues. Alternatively, traditional East Asia medicine has used acupuncture as a treatment for hypertension and may offer a promising approach in response to the limitations of conventional therapy. While detailed clinical and mechanistic experimental evidence is lacking, acupuncture has been observed to reduce BP and improve endothelial function in hypertensive adults. Further, acupuncture has been shown to have specific cerebrovascular effects, increasing cerebrovascular reactivity in healthy adults, highlighting possible neuroprotective properties. Therefore, our review is aimed at evaluating acupuncture as a treatment for hypertension and the potential impact on brain health. We will interrogate the current literature as well as discuss the proposed neural and vascular mechanisms by which acupuncture acts.

## Introduction

Cerebrovascular diseases and neurodegenerative diseases are both leading causes of death in the United States (Xu et al., 2018). Subsequently, with an aging population, the prevalence of cerebrovascular disease and neurodegenerative disease is projected to dramatically increase, raising concerns regarding quality of life and cost of care (i.e., medical expenses, elderly care, nursing fees, etc.) for the American population (Khavjou et al., 2016; Benjamin et al., 2019).

Hypertension independently increases the development of cerebrovascular disease and neurodegenerative disease (Baumgart et al., 2015; Benjamin et al., 2019). Although a direct mechanistic link between hypertension and poor brain health is unknown, having hypertension predicts lower cognitive function (Kilander et al., 1998) and increased risk for dementia with age (Sharp et al., 2011). Many hypothesize that the etiology stems from poor blood pressure (BP) and cerebrovascular regulation. This dysfunction fosters hypoperfusion, stressing the brain through a nutrient mismatch, subtly causing damage over many years (de la Torre, 2012). Animal models indicate that increased systolic BP (SBP) and cerebrovasculature remodeling in hypertension ultimately reduce brain blood flow, leading to behavioral and cognitive impairments (Pires et al., 2013; Wiesmann et al., 2017). In hypertensive adults, a diminished cerebral perfusion may accelerate the development of Alzheimer’s *via* decreased oxygen delivery in ischemia-sensitive brain regions like the hippocampus, inducing neurodegeneration and subsequent cognitive decline (de la Torre, 2000).

Treatment of hypertension has shown benefits in neurodegenerative disease development and overall brain health. Most observational studies have suggested that improved SBP control reduces the risk of Alzheimer’s and other dementias (Qiu et al., 2005; Hughes and Sink, 2016). Additionally, treating hypertension is among the most effective strategies to prevent a stroke (Meschia et al., 2014). Each year, approximately 795,000 US adults suffer a new or recurrent stroke (Benjamin et al., 2019). Therefore, effective treatment of hypertension has tremendous far-reaching impacts on brain health.

However, pharmacological treatment of hypertension faces challenges such as unwanted side effects, limited adherence, and difficulty individualizing treatment for such a diverse population. Up to 97% of patients taking antihypertensive medications experience adverse side effects (Toyoshima et al., 1997; Bardage and Isacson, 2000), which can reduce future adherence (Tedla and Bautista, 2016). Moreover, 25% of patients do not fill their initial antihypertensive prescription (Holland et al., 2008; Franklin et al., 2012; Berra et al., 2016). As a result, four out of five patients do not sufficiently adhere to antihypertensive treatment, failing to control their BP (Petrilla et al., 2005; Gwadry-Sridhar et al., 2013). Additionally, only two out of the six classes of antihypertensive drugs are independently associated with decreased risk of dementia (van Middelaar et al., 2017). These circumstances result in patients maintaining an elevated dementia and stroke risk. Therefore, there is a dire need to identify adequate antihypertensive treatments to improve cerebral blood flow (CBF) that circumvents the limitations presented.

Acupuncture is regarded as a promising complementary and integrative antihypertensive approach that does not share many of the limitations of medical interventions. It is a practice of traditional East Asia medicine in which specific points on the body are stimulated, most often by inserting disposable thin stainless-steel needles through the skin. Acupuncture has documented benefits easing various types of pain (e.g., low back, neck, osteoarthritis, headache, etc.) and conditions including cardiovascular diseases (World Health Organization, 2002; McDonald and Janz, 2017). Western cultures have become more welcoming of acupuncture; a 2017 clinical practice guideline from the American College of Physicians included acupuncture among the nondrug treatment options for management of both acute and chronic back low-back pain (Qaseem et al., 2017). However, acupuncture’s effects on the cardiovascular system are still under-researched, preventing its utilization as a therapeutic option in the Western world.

While detailed clinical and mechanistic experimental evidence is lacking, acupuncture has been observed to reduce BP (Li et al., 2015; Liu et al., 2015) and improve endothelial function in hypertensive adults (Park et al., 2010). Further, acupuncture has been shown to have specific cerebrovascular effects in healthy adults, highlighting possible neuroprotective properties (Byeon et al., 2011; Hyun et al., 2014; Im et al., 2014). However, it is yet to be determined if the literature is in support of using antihypertensive acupuncture prescriptions to improve brain health *via* enhanced cerebrovascular control. Therefore, our review is aimed at evaluating the acupuncture literature; as a treatment for hypertension, its effects on cerebrovascular control, the evidence for specific acupuncture hypertension treatment improving brain health outcomes, and the mechanisms by which acupuncture can affect BP and cerebrovascular control.

## Evidence Supporting Acupuncture as a Treatment of Hypertension

Recent randomized control trials (RCTs) indicate that acupuncture is effective at lowering BP in humans. One of the largest RCTs involving more than 400 mostly non-hypertensive adults found reductions in SBP (122–113 mmHg) and DBP (68–65 mmHg) following 6 weeks of biweekly acupuncture compared to no change in sham and auricular acupuncture (Abdi et al., 2017). However, it should be noted that the auricular acupuncture and the two sham groups had lower initial SBP (110, 116, and 111 mmHg, respectively). This leads to speculation that acupuncture only impacts individuals with higher BP, specifically higher SBP. Therefore, the hypotensive effect of acupuncture may be best represented in hypertensive individuals.

Four weeks of acupuncture treatment (20-minutes/treatment, 2 treatments/week) reduced SBP and DBP ~ 7mmHg in hypertensive adults. After 8 weeks SBP and DBP remained lower (~6.5 and ~4.9mmHg, respectively) than initial values. However, 4 weeks after treatment was ceased, SBP and DBP remained ~5 mmHg lower, with only DBP being significantly different. The time-matched control did not have a change in BP at any time point (Liu et al., 2015). Similarly, Li et al. (2015) compared 8 weeks of acupuncture in hypertensive adults at acupoints shown to have a BP-lowering effect vs. control acupoints (sites that do not alter BP). Acupuncture reduced SBP by 6 mmHg and DBP by 4 mmHg. Subsets of the subjects participated in crossover assessment with the active treatment lowering SBP ~7 mmHg and DBP ~4 mmHg compared to control. A further subset of subjects stopped all treatments and were assessed at 1 and 2 months post-treatment. At 1 month following cessation of treatment, SBP remained lower and DBP reverted to pre-treatment values. Two months after treatment, BP was not different from pre-treatment values. From these studies, it is clear that acupuncture can have an effect on hypertension, and this effect can be sustained for up to 1 month, with diminishing effects thereafter.

The promising results presented above highlight the growing research interest in acupuncture and are only a fraction of the large volume of research that is currently being generated. This volume has further spurred many recent systematic reviews on acupuncture and hypertension. Interestingly, few determine that acupuncture alone can manage BP in hypertensive adults (Wang et al., 2013). Rather, the consensus is that acupuncture, when combined with pharmacological therapy, lowers BP than either treatment alone (Li et al., 2014; Zhao et al., 2015, 2019; Chen et al., 2018; Yang et al., 2018; Niu et al., 2019). Therefore, acupuncture may best be used as an adjunctive treatment for hypertension.

Although the recent results seem promising, it is not unanimous. The above listed reviews were meticulous in limiting bias and incorporating the most robust research, yet there are still methodological concerns with antihypertensive acupuncture RCTs. There is enormous heterogeneity of study design among the RCTs, with no consensus for how to properly provide control/placebo for acupuncture. Control types used in the RCTs include a time-matched group, a sham acupuncture group, and an inactive acupoint acupuncture group. The use of sham acupuncture has been argued as the best means for control (Yang et al., 2018), mainly because the use of “inactive” acupoints raises concerns of possible spillover effects. Meaning, stimulation of an acupoint believed to have no effect on cardiovascular parameters could have a minor effect, limiting statistical determination of active acupoint stimulation. Additionally, many RCTs contain small sample sizes and do not always utilize a crossover design. For instance, in Li et al. (2015), *post hoc* power analysis determined that at least five more participants per group would be needed to achieve the appropriate power. These limitations cause skepticism about the effectiveness, efficacy, and safety of acupuncture treatment in hypertension.

Overall, while it seems that acupuncture’s effect on lowering BP is minimal, as an adjunctive treatment to conventional therapies, acupuncture is a promising avenue in the quest to control hypertension and limit its damaging effects throughout the body. Although meaningful issues are present in most of the RCTs examined, acupuncture appears to be effective in lowering BP in hypertensive adults. However, the lack of mechanistic integrative human research elucidating a link between acupuncture and BP regulation and experimental design concerns justify the need to critically evaluate acupuncture as a reliable treatment option.

## The Effect of Acupuncture Treatment on Cerebral Hemodynamics

Attempts to determine acupuncture’s effect on brain health outcomes have steadily increased over the years. However, true assessment of these outcomes requires longitudinal studies that have yet to be undertaken. Assessing brain hemodynamics and outcomes is more immediate and has been researched using various imaging modalities [e.g., transcranial Doppler (TCD), near-infrared spectroscopy (NIRS), functional magnetic resonance imaging (fMRI), etc.] that have various strengths and weakness that can complicate interpretation. However, each provides valuable information of the brain environment.

Lower CBF, measured using TCD, is associated with cognitive decline and neurodegenerative disease (Ruitenberg et al., 2005). Therefore, it is theorized that treatments that improve or restore CBF may attenuate or possibly prevent the onset of these conditions. Interestingly, TCD studies present evidence that acupuncture can improve CBF and that there is acupoint-cerebral vessel specificity. Acupuncture of GB20 point has been shown to improve CBF regulation in posterior (Vertebral and Basilar) arteries but not the middle cerebral arteries (MCAs; Yuan et al., 1998; Im et al., 2014). Similarly, in healthy adults, a single 20-min acupuncture treatment at acupoint ST36 improved flow and CO_2_ reactivity in the basilar artery but only reactivity in the MCA (Hyun et al., 2014), whereas 20-min acupuncture of GV20 increased CBF and CO_2_ reactivity in both middle and anterior cerebral arteries (basilar not measured; Byeon et al., 2011).

As previously stated, the effects of acupuncture are best exemplified using diseased individuals. Applying acupuncture to stroke patients at acupoints LV3, LV4, SJ5, and GB34 significantly increased CBF (in MCAs). This is accompanied by a decreased SBP; however, the sham acupuncture group also saw a decreased DBP (Ratmansky et al., 2016). Using NIRS, acupuncture intervention on stroke patients showed a significant increase in regional cerebral blood volume or oxyhemoglobin parameters (Li et al., 2011). A systemic review of NIRS studies indicates that acupuncture varies wildly in healthy adults but seems to be more appropriate for disordered populations such as stroke patients (Lo et al., 2015).

Experiments using fMRI have been completed to determine acupuncture’s effect on specific brain regions and the brain network. Acupuncture (Ll11 and ST36) in both healthy and stroke patients improved brain activity in various areas of the brain (Cho et al., 2013). Further, increased activity and connectivity across hemispheres and various portions of the brain following acupuncture has also been observed in patients with mild cognitive impairment (Feng et al., 2012), Alzheimer’s disease (Wang et al., 2014), and stroke (Chen et al., 2014).

Single-photon emission computerized tomography (SPECT) and position emission tomography–computed tomography (PET-CT) allow for a depiction of regional brain perfusion. In healthy subjects, acupuncture (Ll4 and Ll11) increased both regional CBF and glucose metabolism in both frontal regions (An et al., 2009) and specifically at important regions regarding the limbic system, middle cingulum, and medial orbitofrontal gyrus (Jung et al., 2011).

The literature advocates for acupuncture having positive effects on brain hemodynamics in an acupoint-specific manner. However, many of these studies have: (a) been conducted in healthy populations and (b) suffered from similar methodological concerns raised in our discussion of acupuncture on BP regulation. What remains unclear is a mechanistic link between acupoints and brain-specific responses as well as if there is a connection between acupoints used in the treatment of hypertension and cerebral responses. Garnering further understanding of these acupoints and if acupuncture treatment of hypertension actually improves cerebrovascular responses could have a significant biomedical impact as it would provide further information on the mechanistic links between hypertension and poor brain health, as well as offer a greater number of treatment options.

## Effects of Acupuncture Techniques Aimed at Treating Hypertension on Brain Health Outcomes

As alluded to earlier, hypertension is a strong risk factor in the development of cerebrovascular diseases and neurodegenerative diseases. As such, few studies have focused on acupuncture’s effect on stroke *via* regulating hypertension. Work in hypertensive animals support hypertensive-acupuncture treatment that prevents stroke through several pathways related to the nervous system, oxidative stress, the endocrine system, cardiovascular function, and hemorheology (Zheng et al., 2018). However, the human data are unclear. Meta-analyses have challenged the efficacy of acupuncture for the treatment of hypertension as a risk factor for stroke (Sibbritt et al., 2018). However, controlling BP *via* acupuncture as an additive treatment is an effective secondary prevention of stroke (Du et al., 2017). In stroke patients complicated with hypertension, antihypertensive acupuncture improved the National Institutes of Health stroke scale and Barthel index, both of which are indicative of stroke-related neurologic deficit. Further, the treatment reduced morning BP (esp. DBP) and improved SBP and DBP load (Guo and Shi, 2019), therefore suggesting antihypertensive acupuncture as a post-stroke treatment option rather than just a preventative measure.

Studies investigating the possible underlying molecular mechanisms of acupuncture in the treatment of neurodegenerative diseases, specifically dementia, have found that acupuncture can alter neurotrophin regulation (Hwang et al., 2010; Lee et al., 2012; Lin et al., 2016), reduce oxidative damage (Liu et al., 2006), and modulate apoptotic signaling (Chen et al., 2012; Xue et al., 2014) in a manner that promotes positive health outcomes. However, there are few studies that have investigated the effect of acupuncture on dementia directly related to hypertension despite the strong human epidemiological evidence linking the two. Such a study in spontaneous hypertensive rats found that acupuncture at DU20 (also referred to as GV20) and ST36 acupoints reduced BP, increased microvessel dilation, increased CBF, attenuated neuron injury, and restored cognitive impairment (Tian et al., 2013). In hypertensive humans, acupuncture at LR3 activated anterior cingulated gyrus (measured with fMRI) to lower BP through modulation of parasympathetic nervous activity. Additionally, through anterior cingulated gyrus activation, the connection with the surrounding areas was strengthened to improve cognitive impairment caused by long-term hypertension (Sun et al., 2014).

Currently, the evidence of acupuncture’s favorable effect on brain health outcomes directly *via* regulating hypertension is still scant. However, the data are promising and justify more experimental and clinical studies into acupuncture’s reliability as a possible treatment/prevention strategy for brain health outcomes related to hypertension.

## Potential Mechanism(s) of Acupuncture Improving Hypertension and Vascular Control

**Figure 1:**
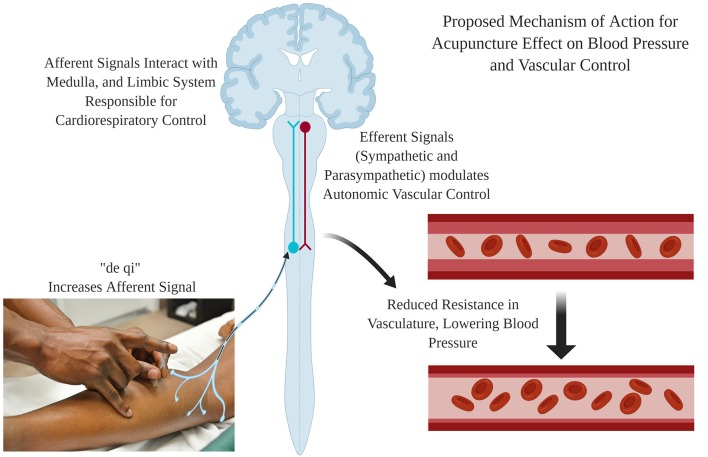
Proposed mechanism of action for the hypotensive effect of acupuncture treatment. Needle insertions and stimulation causes “de qi,” which increases the afferent neuron firing. The afferent signal increases activity in areas of the brain responsible for cardiorespiratory control (Medulla and Limbic System). This activity results in the modulation of autonomic vascular control, reducing vascular resistance and blood pressure (BP). Created using BioRender.

There are many different proposed mechanisms linking acupuncture to positive BP and vascular outcomes; however, most fail at drawing a logical link from acupuncture stimulus to observed outcome. Acupuncture involves sticking needles just below the skin surface, twisting the needle, and expecting a change in a corresponding system such as the cardiovascular system (Figure 1). Accumulating evidence indicates that the hypotensive effect is mediated by a reduction in sympathetic outflow (Sato et al., 1993; Michikami et al., 2006) and an increase in endorphin release (Guo and Longhurst, 2007). Increases in various endorphins has independently been linked to reduced BP in hypertension (Bądzyńska et al., 2016; Li et al., 2016). This seems to be a paradox, as previous research shows venipuncture increases catecholamine release, synonymous with a hypertensive effect (Frankenhaeuser et al., 1976). The difference must be attributed to the depth of the needle (venous vs. just below the skin surface) and the twisting of the needle, which promotes “de qi,” a key component of acupuncture. Langevin et al. (2001) describe that, during de qi, the connective tissue is wrapped around the needle, promoting tension. The authors speculate that this tension could be activating various sensory organs within the skin, increasing afferent nerve firing. Zhou et al. (2005a) specifically looked at responses to acupoints that reduced BP and observed increased afferent sensory neuronal firing. When afferent nerves are severed, the hypotensive effect and sympathetic nerve inhibition are eliminated (Sato et al., 1993). In healthy humans, the data are not definitive. Stimulation of acupoints PC6 and HT7 have been argued to elicit hypotensive effects through alterations in sympathetic (Jung et al., 2006) and parasympathetic nerve activity (Jung et al., 2007). Conversely, in patients with post-stroke insomnia, intradermal acupuncture at PC6 and HT7 greatly decreased the number of non-dippers possibly by lowering LF/HF ratio (Lee et al., 2009). Taken together, acupuncture works through a feedback loop where afferent nerves initiate a correction in autonomic nerve outflow, properly regulating BP. Insight into the mechanisms by which acupuncture is reducing sympathetic outflow come from elegant animal studies investigating the activity in the rostral ventrolateral medulla (RVLM) where sympathetic nerve activity is controlled. In normotensive rats, acupuncture (PC6) may alter the baroreflex. PC6 stimulation increased afferent neuron firing, which was associated with a decrease in RVLM activity, resulting in a reduction of sympathetic outflow and a lowering of BP (Zhou et al., 2005b). Similarly, Wang et al. (2018) performed a highly controlled study proving that acupuncture (LR3) reduces oxidative stress in the RVLM and that the observed antihypertensive effect was directly tied to NADPH oxidase activity and the REDOX environment of the RVLM in spontaneously hypertensive rats. Again, linking reduced BP and sympathetic outflow *via* RVLM. Alternatively, in humans, acupuncture increases firing in the gracile nucleus, frontal lobe, cerebellum insula, hypothalamus, and many other areas of the brain related to maintenance of BP (Chen and Ma, 2003; Chen et al., 2013; Zheng et al., 2016).

While the cited research has provided a general mechanistic outline for acupuncture to modulate BP, these data are far from conclusive. However, the promising results provide the foundation for future research to delineate physiological mechanistic responses to acupuncture. Until these mechanisms are well understood and verifiable, mass adoption of acupuncture as an additive treatment for hypertension cannot be recommended.

## Limitations

This review is not without some limitations. First, this review prioritized human RCTs. Thus, the volume of studies reviewed and the sample size within each reviewed article are limited. However, we contest that RCTs provide the best evidence of the effectiveness of acupuncture (Sibbald and Roland, 1998). Further, this choice limits review of articles using animal models especially outside our “mechanisms” section. Thus, some well-designed studies were omitted. However, these restrictions allow for the most rigorous and translational review regarding acupuncture’s effect on BP and cerebrovascular control. Finally, mostly English language articles were cited. Acupuncture literature is written in many other languages and we have included several non-English articles (Li et al., 2011; Sun et al., 2014; Zheng et al., 2018; Guo and Shi, 2019). However, there may be a few relevant articles that our study team was unable to access.

## Discussion

Evaluation of the literature suggests that acupuncture, at best, can be used as a co-treatment option for hypertension. Further, acupuncture has been found to improve cerebrovascular control and brain activation in regions consistent with positive health outcomes *via* the maintenance of BP and cognition. The mechanisms that link acupuncture to positive health outcomes are still poorly understood, but they appear to be linked to improvements in autonomic cardiovascular control. Regardless, the evidence is in support of safe and efficacious use of acupuncture in human hypertension. Yet, there is a profound need for tightly controlled mechanistic human research to determine the validity and the physiological underpinnings of acupuncture before wider adoption of acupuncture as a treatment option for improved cardiovascular and brain health can be recommended.

## Author Contributions

JS, JA, and JK contributed equally to the writing and editing of the manuscript.

## Conflict of Interest Statement

The authors declare that the research was conducted in the absence of any commercial or financial relationships that could be construed as a potential conflict of interest.
